# Structure and extent of DNA methylation-based epigenetic variation in wild emmer wheat (*T. turgidum* ssp. *dicoccoides*) populations

**DOI:** 10.1186/s12870-015-0544-z

**Published:** 2015-08-14

**Authors:** Anna Venetsky, Adva Levy-Zamir, Vadim Khasdan, Katherine Domb, Khalil Kashkush

**Affiliations:** Department of Life Sciences, Ben-Gurion University, Beer-Sheva, 84105 Israel

**Keywords:** Emmer wheat, DNA methylation, Transposable elements, Biodiversity

## Abstract

**Background:**

The genetic structure and differentiation of wild emmer wheat suggests that genetic diversity is eco-geographically structured. However, very little is known about the structure and extent of the heritable epigenetic variation and its influence on local adaptation in natural populations.

**Results:**

The structure and extent of the heritable methylation-based epigenetic variation were assessed within and among natural populations of *Triticum turgidum* ssp*. dicoccoides*. We used methylation sensitive amplified polymorphism (MSAP) and transposon methylation display (TMD) techniques, to assess the methylation status of random genomic CCGG sites and CCGG sites flanking transposable elements (TEs), respectively. Both techniques were applied to the DNA of 50 emmer accessions which were collected from five different geographically isolated regions. In order to ensure the assessment of heritable epigenetic variation, all accessions were grown under common garden conditions for two generations. In all accessions, the difference in methylation levels of CCGG sites, including CCGG sites that flanked TEs, were not statistically significant and relatively high, ranging between 46 and 76 %. The pattern of methylation was significantly different among accessions, such that clear and statistically significant population-specific methylation patterns were observed.

**Conclusion:**

In this study, we have observed population-unique heritable methylation patterns in emmer wheat accessions originating from five geographically isolated regions. Our data indicate that methylation-based epigenetic diversity might be eco-geographically structured and might be partly determined by climatic and edaphic factors.

**Electronic supplementary material:**

The online version of this article (doi:10.1186/s12870-015-0544-z) contains supplementary material, which is available to authorized users.

## Background

Emmer wheat (*Triticum turgidum* ssp*. dicoccoides*) is an allotetraploid species, which harbors of two different genomes (AA and BB), and is distributed over the near east Fertile Crescent [1, 2]. Emmer wheat is the wild progenitor of emmer (*T. turgidum* ssp*.* dicoccum), from which all *T. turgidum* ssp*. durum* (pasta wheat) and *T. aestivum* (bread wheat) were derived. While crop yields have recently increased for the most part, the genetic basis of most of the important food crops has been rapidly narrowing [3]. This is due to the global extension of modern pure breeding practices, which increase genetic homogeneity [4]. The loss of genetic diversity of some of the world’s crops has accelerated greatly in recent decades, with many crops becoming increasingly susceptible to diseases, pests and environmental stresses. Wild cereals are widely adaptive to all these stressful factors. This explains why wild relatives of cultivated wheat, and in particular wild emmer wheat, *T. turgidum* ssp. *dicoccoides* (the mother of wheat), have been of great interest to crop researchers and the subject of extensive research in the past few decades.

Previous works investigating the genetic structure and differentiation of wild emmer wheat suggest that genetic diversity is eco-geographically structured and might be partly determined by climatic and edaphic factors [5–11]. A previous study on emmer wheat populations in micro-geographic sites in Israel, using allozymes and random amplified polymorphic DNA (RAPD) markers, showed a possible nonrandom adaptive genetic differentiation at single and multilocus levels in contrasting soils, topographies, and climate [6]. Discriminate analyses using allozyme markers differentiated between one central and three marginal regions, as well as between different soil-types within the populations in Israel [10]. In addition, a strong SSR diversity was found among three populations and two edaphic (soil type) groups of *T. dicoccoides* [6]. It was suggested that SSR variation is influenced by both genetic factors and ecological forces [6]. Although much genetic research had been conducted over the years, none of the studies attempted to explain the phenotypic polymorphism by examining epigenetic factors, such as cytosine methylation. The observed genetic variation between and within wild emmer wheat populations was significantly higher than the reported genetic variation in cultivated wheat [12]. While the observed genetic variation (of DNA markers) in most cases might be neutral, namely it might not impact genomic function, epigenetic variation could have a direct impact on genome function, and through this might affect the fitness of an organism to specific environmental conditions.

Epigenetic regulation is the heritable alteration of the extent of gene products by modifications other than in the DNA sequence. It consists mostly of 5-cytosine methylation at CG and CHG sites [13]. As a general rule, hypermethylation is correlated with down-regulation of gene expression, while hypomethylation is correlated with up-regulation of gene expression [14]. The bias of methylation toward repetitive DNA suggests that silencing transposable elements (TEs) is one of the primary roles of DNA methylation [15]. The *Arabidopsis* genome contains 24 % methylated CG sites, 6.7 % methylated CHG sites (H = A, C or T) and 1.7 % methylated CHH sites [16]. All transposable element sequences are usually methylated in *Arabidopsis*, in all sequence contexts [15]. Considering that DNA demethylation or methylation of transposable element sequences is associated with their activation or silencing, respectively, TEs are hypermethylated compared to host genes in plants [17–19]. Thus, there is an increased interest in understanding the role of epigenetic processes in ecology and evolution. However, almost nothing is known on the structure and extent of methylation-based epigenetic variation in wild plant populations in general, and in wheat populations in particular. Using the methylation-sensitive amplified polymorphism (MSAP) assay [20, 21], two studies reported on the structure and amount of methylation variation at CCGG sites in wild populations of barley [9] and *Viola cazorlensis* [22]. To date, there are no reports on extensive studies on the structure and amount of epigenetic variation in natural populations of wild emmer wheat.

In this study we aimed to assess the epigenetic biodiversity, through cytosine methylation, within and between populations of wild emmer wheat using accessions collected from five geographically isolated regions with different climatic conditions such as: rainfall level, humidity, soil type and biotic conditions [23]. More specifically, we have assessed: (1) the structure and amount of cytosine methylation variation at CCGG sites in a genome-wide manner, using MSAP assay; and (2) the structure and amount of cytosine methylation variation at CCGG sites flanking transposable elements, using the TMD assay [24]. To this end, we observed statistically significant population-unique heritable methylation patterns. The possible adaptive value of the observed epigenetic variations in wild emmer wheat is discussed.

## Results

### Genome-wide analysis of DNA cytosine methylation of CCGG sites

**Fig. 1:**
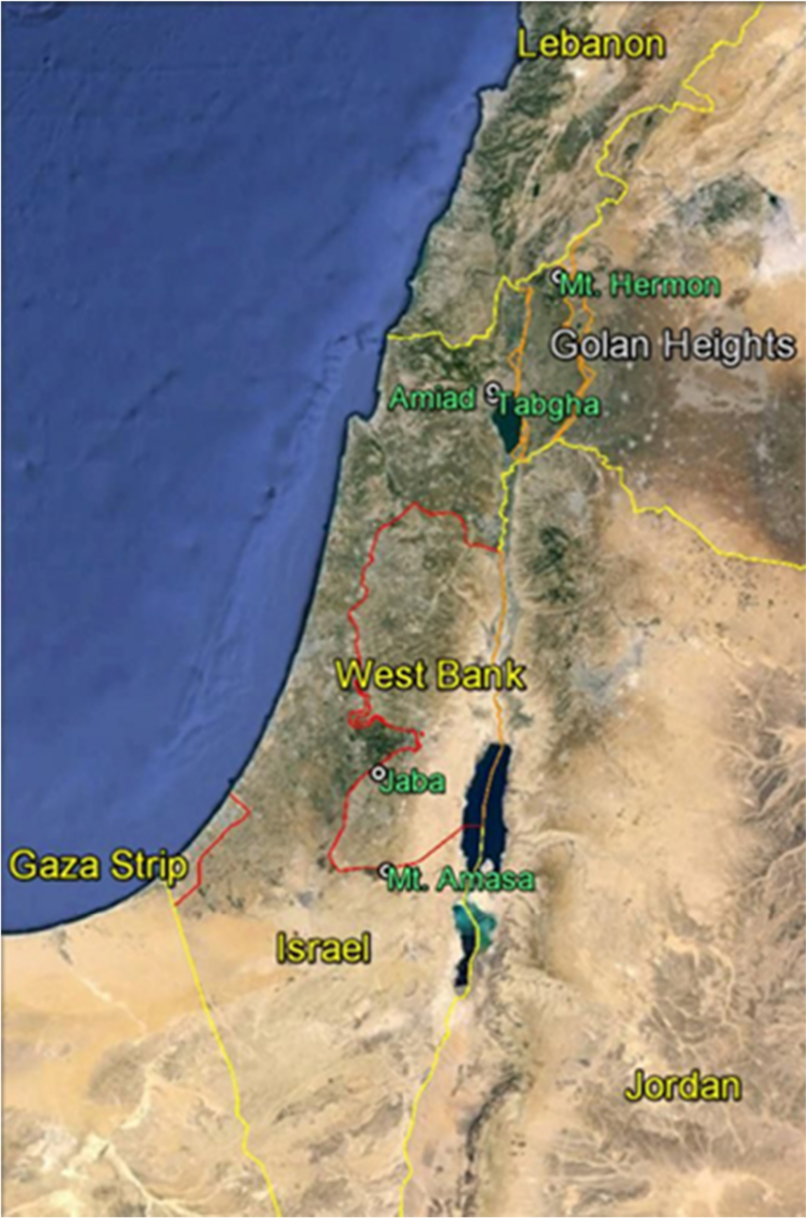
A map of Israel and the surrounding areas of the West Bank, Gaza strip and parts of Jordan, Lebanon and Syria. The five collection sites (Mount Hermon, Amiad, Tabgha, Jaba and Mount Amasa) of wild emmer wheat are indicated in green. This map was created in Google Earth. See Additional file 1: Table S1 for more details on the ecogeographical nature of the collection sites

It is known that methylation patterns in plants can be inherited over generations [22]. This heritable epigenetic variation might have an evolutionary role in adaptation and divergence of natural populations. In order to reduce temporal methylation variations among accessions in the different populations we have synchronized the growth of all plants collected from the five populations in the same greenhouse (common garden). Figure 1 describes the location of the five collection sites (Mount Hermon, Amiad, Tabgha, Jaba and Mount Amasa) and the ecogeographical data (including altitude, annual rainfall, mean annual temperature and soil type) of all five collection sites are described in Additional file 1: Table S1. DNA was extracted from young leaves (one month post germination) from all accessions and was subjected to MSAP analysis. The analysis is based on the cleavage patterns of two enzymes, *Hpa*II and *Msp*I, which both cleave unmethylated CCGG sites. *Msp*I (but not *Hpa*II) cleaves when the internal cytosine is methylated (CG methylation status), while *Hpa*II (but not *Msp*I) cleaves when the external cytosine is methylated (CHG methylation status) only when the methylation occurs in one strand (hemi-methylation) [25]. The level of methylation for each individual can be measured by the number of sites with polymorphic bands between the *Msp*I and *Hpa*II MSAP reactions in the same individual out of the total number of MSAP sites. Examples of radioactively-labeled and fluorescently-labeled MSAP patterns are shown in Additional file 2: Figure S1. To this end, 447 reproducible MSAP sites were analyzed in all 50 accessions. It is important to mention that the accessions which showed low quality MSAP patterns were excluded from the analysis and that polymorphic bands which could correspond to typical AFLP variation (genetic) were excluded from the analysis. Namely, for each site, only variation which had originated from cytosine methylation (polymorphism between the *Msp*I and *Hpa*II MSAP reactions) was considered.

The average level of methylation was measured in all five populations and found to be statistically similar (Additional file 2: Figure S2): 65.2 % in Mt. Hermon, 66.8 % in Amiad, 63.7 % in Tabgha, 65 % in Jaba and 71.3 % in Mt. Amasa. However, for four populations (Mt. Hermon, Amiad, Tabgha and Jaba), the context of methylation in a majority of the sites (62.3 %, 59.9 %, 65.2 % and 60.2 %, respectively) occurred in CHG positions (bands present in H lanes only), while for the Mt. Amasa population, the level of CHG methylation was similar to the level of CG methylation. Similarly, the methylation levels in the genome of the three *T. dicoccoides* accessions from Turkey, Iran and Syria were 76.4 %, 66.7 % and 62 %, respectively. Note that we cannot conclude that the methylation level in the Turkish *T. dicoccoides* is significantly higher because only one accession was tested.

**Fig. 2:**
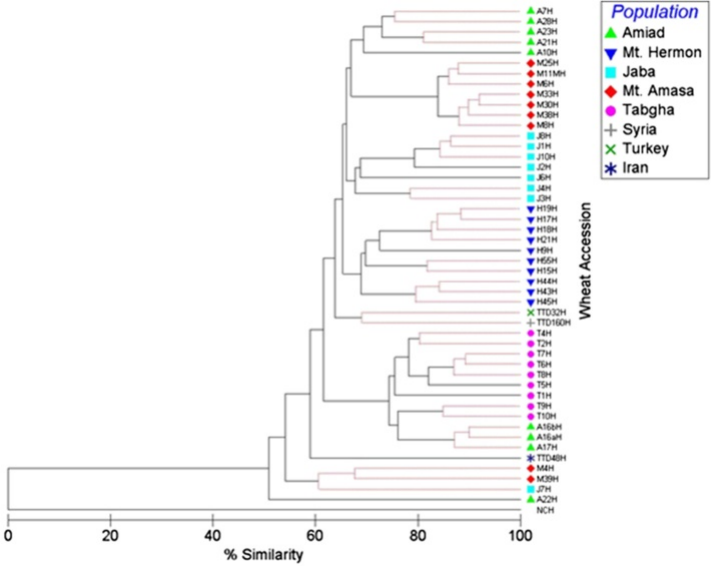
Phylogenetic tree generated by multi-dimensional scaling using 447 MSAP bands from accessions of five populations: Mt. Hermon, Amiad, Tabgha, Jaba and Mt. Amasa. Accessions TTD48, TTD32 and TTD16 were collected from Syria, Iran and Turkey, respectively, and were used as outsider controls in this analysis. The index (top right) indicates the collection site of each one of the 53 accessions. NCH, at the bottom of the phylogenetic tree indicates a negative control (water was used as a template in MSAP reaction). The black lines indicate significant separation, while red lines indicate insignificant separation. The level of epigenetic similarity is indicated on bottom. See Additional file 2: Figure S3 for more details on the statistical analysis

A phylogenetic tree was built based on the methylation patterns of the 447 CCGG sites from MSAP, for 44 accessions (Fig. 2). The phylogenetic tree significantly clustered the accessions (*p* < 0.05, global R = 0.638, pairwise R > 0.3) based on their geographical origin (populations). In Fig. 2 it can be seen that accessions from Mt. Hermon were significantly clustered in one group based on their methylation patterns, and so were Jaba, Mt. Amasa and Tabgha accessions (Additional file 2: Figure S3). The Amiad accessions were clustered in two main groups, the first group contained 5 accessions, while the second group, which is similar to the Tabgha cluster, contained three accessions. This might indicate a high level of epigenetic variation in the Amiad population. One explanation is that the collection from the Amiad site was from a relatively large area and it was previously reported on the wide variation within this population [23]. The *T. dicoccoides* accessions from Turkey and Iran were significantly clustered in one group based on their CHG methylation status. Interestingly, the Syrian accession was similar to the Mt. Hermon cluster, which is geographically closer.

We have randomly extracted 15 bands from radioactively-labeled MSAP (from the Tabgha accessions), reamplified and sequenced them (Additional file 3: Table S1). All sequences were used as queries in plant sequence databases (see materials and methods) and 10 out of the 15 sequences hit transposable elements, while the remaining 5 sequences did not hit annotated genes or non-coding sequences. Transposable elements are considered key players in organismal evolution because they play a prominent role in genomic rearrangements [26, 27]. Here we have assessed the contribution of two transposable element families, *Veju* (a TRIM retrotransposon) and *Thalos* (a MITE from the *Tc1/Mariner Stowaway*-like superfamily) to the methylation-based epigenetic variation in wild emmer wheat populations.

### Analysis of the methylation patterns of CCGG sites flanking *Veju* elements

It is known that in plants, TEs are often targeted for methylation, as such they are said to be hypermethylated compared to other genomic sequences [18]. Recently, it was observed that the methylation surrounding TEs was significantly higher than the methylation of random genomic sequences [28, 29]. To this end, the level of methylation in *Veju*-flanking CCGG sites was measured for each one of the accessions and then the average methylation level was calculated for each population (Additional file 2: Figure S4). It is important to mention that polymorphic bands among accessions that could be the result of a transposition event and did not show any methylation changes (polymorphism between the *Msp*I and *Hpa*II TMD reactions) were excluded from the analysis. However, some of the polymorphic sites that showed methylation changes could be the results of polymorphism in the TE insertion sites.

Based on the analysis of 290 TMD bands, the average level of methylation of CCGG sites flanking *Veju* was: 51.3 % in Mt. Hermon, 52.8 % in Amiad, 46.5 % in Tabgha, 50.9 % in Jaba and 48.3 % in Mt. Amasa. The average methylation levels among populations were statistically similar (Additional file 2: Figure S4). In addition, the methylation levels in the genome of the *T. dicoccoides* accessions from Turkey and Iran were 55.3 %, and 63.5 %, respectively.

**Fig. 3:**
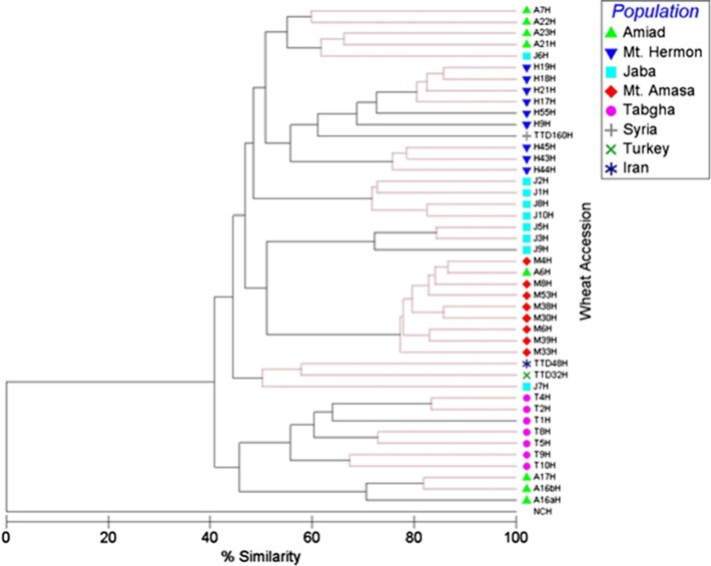
Phylogenetic tree generated by multi-dimensional scaling using 290 TMD bands corresponding to *Veju*-CCGG flanking sites, from accessions of five populations (see top right index). NCH, at the bottom of the phylogenetic tree indicates a negative control (water was used as a template in MSAP reaction). The black lines indicate significant separation, while red lines indicate insignificant separation. The level of epigenetic similarity is indicated on bottom. See Additional file 2: Figure S5 for more details on the statistical analysis

The resulting phylogenetic tree significantly clustered the accessions (*p* < 0.05, global R = 0.651, pairwise R > 0.3) based on their geographical origin (Fig. 3). Accessions from Mt. Amasa were significantly clustered in one group based on their methylation patterns, as were Mt. Hermon, Tabgha and Jaba accessions (Additional file 2: Figure S5), while accessions from Amiad were clustered in two main groups, the first group contained 3 accessions, while the second group (also containing three accessions) was clustered close to the Tabgha population. Furthermore, the *T. dicoccoides* accessions from Turkey and Iran were clustered in one group, while the Syrian accession was clustered in the Mt. Hermon group.

### Analysis of the methylation patterns of CCGG sites flanking *Thalos* elements

**Fig. 4:**
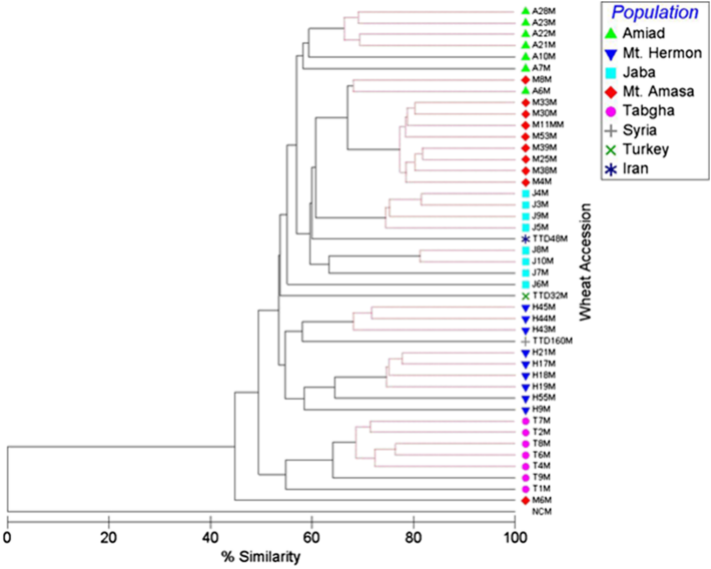
Phylogenetic tree generated by multi-dimensional scaling using 401 TMD bands corresponding to *Thalos*-CCGG flanking sites from accessions of five populations (see top right index). NCM indicates a negative control. The black lines indicate significant separation, while red lines indicate insignificant separation. The level of epigenetic similarity is indicated on bottom. See Additional file 2: Figure S7 for more details on the statistical analysis

Based on the analysis of 401 TMD bands, the average level of methylation of CCGG sites flanking *Thalos* was statistically similar among populations (Additional file 2: Figure S6): 60.1 % in Mt. Hermon, 55.6 % in Amiad, 50.1 % in Tabgha, 51 % in Jaba and 57.7 % in Mt. Amasa. Furthermore, the methylation levels in the genome of the *T. dicoccoides* accessions from Iran and Syria were 52.9 and 57.9 %, respectively. The phylogenetic tree significantly clustered the accessions (*p* < 0.05, global R = 0.642, pairwise R > 0.3) based on their geographical origin (Fig. 4). Accessions from Mt. Hermon were significantly clustered in one group based on their methylation patterns, as were Jaba, Mt. Amasa and Tabgha accessions (Additional file 2: Figure S7). In addition, Amiad accessions were significantly clustered in one main group containing 6 out of the 10 accessions (see Additional file 2: Figure S7). Furthermore, the *T. dicoccoides* accessions from Turkey, Iran and Syria were clustered in one group based on their methylation patterns.

## Discussion

In this study, we have performed genome-wide analyses of cytosine methylation of CCGG sites in the genomes of wild emmer wheat accessions collected from five geographically isolated regions. More specifically, we performed an analysis of random and TE-flanking CCGG sites. We found that variations in the cytosine methylation are relatively high and observed population-specific epigenetic patterns based on geographical region.

We have analyzed the methylation status of 447 CCGG sites in the genome of 50 accessions of wild emmer wheat from five geographically isolated populations, using an unbiased assay – MSAP. We observed that 63.7–71.3 % of those CCGG sites were methylated in all accessions, indicating a relatively high fraction of heritable methylation patterns in wild emmer compared to domesticated *T. turgidum* species (~35 % methylation in durum wheat, [25]). When the methylation patterns were compared among the 50 accessions, most of the accessions were significantly clustered based on their geographical location, suggesting that accessions in each population might have adapted unique patterns of inherited cytosine methylation. Another possibility is that the population-specific methylation patterns might have been the result of a founder effect in each population. However, in some cases, accessions from one population were similar in their methylation patters to accessions from other populations. Similarly, and using the same methodology as in our study, population-specific methylation patterns were observed in wild populations of *V. cazorlensis* [22]. Importantly, in our study the methylation patterns were assessed in the second generation under common garden conditions (spikes of each plant were bagged to ensure self-pollination), and the results were very similar to those observed in the first generation, indicating that the observed population-specific methylation patterns were inherited. It is important to mention that the use of common garden conditions allows us to ensure not only the assessment of the heritable methylation patterns, but also the accuracy of the statistical analysis that were performed on the methylation data, although in some cases the common garden conditions would be different from the natural conditions for some populations. Hence, a common garden might in fact cause minor epigenetic changes, but this should not affect the veracity of the conclusion since the common garden conditions are not stressful to any of the populations. The key question is whether this epigenetic differentiation of populations is associated with adaptive genetic divergence, because unlike the natural DNA sequence variation-based markers, methylation-based variation might affect genome function by altering gene expression. In order to have some hint about the type of sequences that might be targeted for methylation, we have randomly sequenced and annotated 15 MSAP bands that showed methylation alteration among accessions in different populations and found that most of them (10 of the 15) corresponded to transposable elements, indicating that TEs are massively targeted for methylation and might be differentially affected by epigenetic factors in different populations (population-unique methylation patterns).

### Epigenetic variation adjacent to transposons

Here we have analyzed the methylation status adjacent to two TE families: *Veju* (a TRIM retrotransposon) and *Thalos* (a MITE from the *Tc1/Mariner Stowaway*-like superfamily), using the TMD assay. The analysis included a random subset of *Veju* and *Thalos* insertions (CCGG sites flanking 290 and 401 elements, respectively). Although there are no reports on the exact copy number of either *Veju* or *Thalos* families in emmer wheat, our estimation is that they include hundreds to thousands of copies in the wheat genome (data not shown and [30], respectively). Similar to the MSAP results, TMD showed that the methylation levels of CCGG sites flanking the two TE families in wild *T. turgidum* (wild emmer) seem to be higher than the methylation levels in domesticated *T. turgidum* (durum). The average methylation level of CCGG sites flanking *Veju* in wild emmer wheat is ~50 %, while the average methylation level in domesticated durum is ~40 % [31]. The average methylation level of CCGG sites flanking *Thalos* in wild emmer wheat is ~54 %, while the average methylation level in domesticated durum is ~36 % [29]. A previous study in plants showed that in model plant systems, the methylation levels of transposons are significantly higher than the methylation levels of other genomic regions [18]. This observation was corroborated when we assessed the methylation levels in domesticated wheat species [25, 29, 31]. However, in this study we observed that the methylation levels in genomic regions were even higher than the methylation status around TEs, indicating that epigenetic factors might play a major role not only in regulating TE activity, but also in regulating other functional sequences in natural populations. Furthermore, we observed population-specific methylation patterns of CCGG sites around *Veju* and *Thalos*, indicating that the epigenetic regulation of TEs might be specific to local environmental conditions. The population-specific patterns were also observed in the second generation under common garden conditions.

## Conclusions

In this study, we used MSAP and TMD techniques to assess the structure and extent of methylation-based epigenetic variation in natural populations of wild emmer wheat. We observed a relatively high level of heritable methylation at CG and CHG sites in wild emmer wheat. Note that similar phylogenetic trees were observed when CG or CHG sites were analyzed separately. On average, over 50 % of the tested CCGG sites (for both MSAP and TMD assays) were constantly methylated over two generations under common garden conditions. This observed level of methylation is underestimated because both assays detect methylation only when one of the two cytosines at a CCGG site is methylated, whereas if both cytosines are methylated, both enzymes will not cleave the site and discrimination between methylation and typical genetic polymorphisms is difficult. This study provides hints on the important role of DNA methylation and transposable elements on adaptive genetic divergence in wild emmer wheat populations. Future studies will allow assessment of the potential of population-specific methylation patterns to differentially affect gene function under varying environmental conditions.

## Methods

### Plant material

A collection of wild emmer (*T. turgidum* ssp. *dicoccoide*s) from five geographically isolated sites in Israel was used (Fig. 1): Mount Hermon, Amiad, Tabgha, Jaba and Mount Amasa. Seed material (10-40 accessions from each population) was kindly provided by Dr. Sergei Volis from Ben-Gurion University. Plants (accessions) from each population were grown in a greenhouse under similar conditions (common garden). We obtained additional seeds from Turkey, Iran and Syria for comparison, which were kindly provided by Prof. Moshe Feldman from The Weizmann Institute of Science. Leaf material was harvested approximately 4 weeks post germination for DNA extraction [using the DNeasy plant mini kit (QIAGEN)].

### MSAP (methylation-sensitive amplified polymorphism)

MSAP is a modification of the typical AFLP assay described previously [32]. MSAP involves two isoschizomers [20, 21], *Hpa*II and *Msp*I, which both cut unmethylated CCGG sites. While *Hpa*II is sensitive (does not cut) if one or both cytosines are methylated, *Msp*I cleaves when the internal cytosine is methylated. In case of hemimethylation (only one strand is methylated) of the external cytosine, *Hpa*II will cut but not *Msp*I [25]. In this study, we follow the protocol provided by Shaked et al. [25] that was established for analysis of the wheat genome. In an MSAP pattern, monomorphic bands between the *Hpa*II and *Msp*I digested DNA templates (from the same DNA sample) indicate unmethylated CCGG sites, while polymorphic bands indicate methylated sites. The level of methylation for each individual can be measured based on the number of polymorphic bands between the *Msp*I and *Hpa*II MSAP reactions in the same individual, as the number of polymorphic bands out of the total number of MSAP bands. The methylation status of over 200 CCGG sites can be screened in one fluorescently-labeled MSAP reaction and over 70 CCGG sites in one radioactively ^32^P-labeled MSAP reaction. In this study two primer combinations were used in the fluorescent MSAP: a fluorescently-labeled *Hpa*II/*Msp*I primer (CATGAGTCCTGCTCGGTCAG), together with each one of the *EcoR*I primers (GACTGCGTACCAATTCACG and GACTGCGTACCAATTCAAC). In order to extract MSAP bands of interest we performed one radioactively ^32^P-labeled MSAP reaction, with a *Hpa*II/*Msp*I primer (CATGAGTCCTGCTCGGTCAG) and an *EcoR*I primer (GACTGCGTACCAATTCACG).

### TMD (Transposon methylation display)

TMD allows the analysis of the methylation status of CCGG sites in sequences flanking TEs in a genome-wide manner. The method was carried out as previously published [24, 29, 30, 33]. The assay involves the use of one TE-specific primer and another primer complimentary to the adaptor sequence core of the *Hpa*II/*Msp*I site. Thus, each TMD band is a chimeric sequence (TE/flanking DNA). In this study we have analyzed the methylation status of CCGG sites flanking two TE families: (1) a miniature inverted-repeat transposable element (MITE), called *Thalos* (class II) [29]; and (2) a terminal inverted repeat in miniature (TRIM) LTR retrotransposon (class I), called *Veju* [34, 35]. Fluorescently-labeled primers from *Thalos* (GCTCCGTATGTAGTCACTTATTGA) and *Veju* (GACGGTATGCCTCGGATTTA) termini were used together with *Hpa*II/*Msp*I primer (CATGAGTCCTGCTCGGTCAG).

### Constructing of phylogenetic trees

Radioactively labeled selective PCR products of MSAP were electrophoresed on a 6 % polyacrylamide gel, and then exposed to an X-ray film. The fluorescently-labeled MSAP and TMD reactions were electrophoresed in a 3730xl DNA analyzer (Applied Biosystems) and the analyzed using GeneMapper v4.0 (Applied Biosystems). The MSAP and TMD bands were used to create an excel table summarizing the presence (1) or absence (0) of each band (allele) at each site in all samples. Hierarchical agglomerative clustering analysis of the data with Bray-Curtis similarity and construction of the dendrogram (phylogenetic trees) was performed using the Primer6 software version 6.1.6 [Primer-E; [36]]. The similarity profile (SIMPROF) test was used on each node to assess the statistical significance of the dendrogram. SIMPROF calculates a mean profile by randomizing each variable’s values and re-calculating the profile. The pi statistic is calculated as the deviation of the actual resemblance profile of the resemblance matrix with the mean profile. This is compared with the deviation of further randomly-generated profiles to test for significance. To this end, in each phylogenetic tree, statistically significant clusters are indicated by black lines and insignificant clusters are indicated by red lines.

Additional statistical analyses, using Primer6 software, were performed to test the statistical significance of MSAP or TMD patterns between groups (clusters). A resemblance matrix using the Jaccard similarity measure was constructed, and then performed analysis of non-metric Multi-Dimensional Scaling (MDS) and similarity (ANOSIM) between defined populations. MDS produces an ordination based on a distance or dissimilarity matrix where similar groups are clustered on a two dimensional plot, and ANOSIM uses permutation/randomization methods to test for differences between groups to produce *p*-values of the significance of separation, and global and pairwise R statistics of the strength of separation (while R > 0.3 indicates significant separation, R values ranged between 0 and 1).

### Sequence analysis

The derived sequences were annotated using EST and mRNA databases from PlantGDB (http://www.plantgdb.org/prj/ESTCluster/) and NCBI (http://www.ncbi.nlm.nih.gov/nucest/).

### Availability of supporting data

Data was deposited in Dryad: DOI: doi:10.5061/dryad.g31cv

## Additional files

Additional file 1: Table S1. Ecogeographical data for five wild emmer wheat populations in Israel. Additional file 2:
**Figure S1.** Examples of MSAP banding patterns in wild emmer wheat accessions. (**A**) Radioactively-labeled MSAP patterns of three wild emmer wheat accessions. The DNA of each one of the accessions (samples) was cleaved either with the *Hpa*II (H lane) or *Msp*I (M lane) restriction enzyme. In each DNA sample, monomorphic bands between H and M lanes (black arrow) indicate that the CCGG site is unmethylated, while polymorphic bands (red arrow) indicate methylated sites. The methyl ation level for each accession is measured by dividing the total number of polymorphic sites (between H and M lanes) by the total number of sites. Note that monomorphic bands were scored only once. (**B**) Fluorescently-labeled MSAP patterns showing the H and M lanes of one of the wild emmer wheat accessions. The peak position (X axis) indicates the PCR product size of each band. The peak height (Y axis) indicates the band intensity, which has no merit in this qualitative analysis. The MSAP products were electrophoresed in a 3730xl DNA analyzer (Applied Biosystems) and analyzed using GeneMapper v4.0 (Applied Biosystems). All peak presence data were transferred to an excel file for further analysis. **Figure S2.** Average level of cytosine methylation in CCGG sites as assessed by MSAP in five wild emmer wheat populations (10 accessions in each population, indicated by diffirent colors). Standard errors are indicated. **Figure S3.** Non-metric Multi-Dimensional Scaling (MDS) anlaysis using the Jaccard similarity measure in Primer6 software for the MSAP analysis. MDS produces an ordination based on a distance or dissimilarity matrix where similar groups are clustered on a two dimensional plot. The index on the right top indicates the different groups (populations). The calculated *p*-values among the different groups are: 0.001 between Mt. Hermon and Amiad, 0.02 between Jaba and Amiad, 0.002 between Jaba and Mt. Hermon, 0.004 between Mt. Amasa and Amiad, 0.001 between Mt. Amasa and Mt. Hermon, 0.004 between Mt. Amasa and Jaba, 0.004 between Tabgha and Amiad, 0.001 between Tabgha and Mt. Hermon, 0.004 between Tabgha and Jaba, and 0.004 between Tabgha and Mt. Amasa. **Figure S4.** Average level of cytosine methylation in CCGG sites flanking *Veju* retrotransposon as assessed by TMD in five wild emmer wheat populations (10 accessions in each population, indicated by diffirent colors). Standard errors are indicated. **Figure S5.** Non-metric Multi-Dimensional Scaling (MDS) anlaysis using the Jaccard similarity measure in Primer6 software for TMD analysis of *Veju* retrotransposon. The index on the right top indicates the different groups (populations). The calculated *p*-values among the different groups are: 0.004 between Mt. Hermon and Amiad, 0.1 between Jaba and Amiad, 0.004 between Jaba and Mt. Hermon, 0.03 between Mt. Amasa and Amiad, 0.004 between Mt. Amasa and Mt. Hermon, 0.008 between Mt. Amasa and Jaba, 0.2 between Tabgha and Amiad, 0.009 between Tabgha and Mt. Hermon, 0.009 between Tabgha and Jaba, and 0.02 between Tabgha and Mt. Amasa. **Figure S6.** Average level of cytosine methylation in CCGG sites flanking *Thalos* DNA-transposon as assessed by TMD in five wild emmer wheat populations (10 accessions in each population, indicated by diffirent colors). Standard errors are indicated. **Figure S7.** Non-metric Multi-Dimensional Scaling (MDS) anlaysis using the Jaccard similarity measure in Primer6 software for TMD analysis of *Thalos* DNA transposon. The index on the right top indicates the different groups (populations). The calculated *p*-values among the different groups are: 0.009 between Mt. Hermon and Amiad, 0.02 between Jaba and Amiad, 0.004 between Jaba and Mt. Hermon, 0.1 between Mt. Amasa and Amiad, 0.001 between Mt. Amasa and Mt. Hermon, 0.02 between Mt. Amasa and Jaba, 0.06 between Tabgha and Amiad, 0.009 between Tabgha and Mt. Hermon, 0.02 between Tabgha and Jaba, and 0.005 between Tabgha and Mt. Amasa. Additional file 3: Table S1. Molecular characterization of isolated MSAP bands.

## Abbreviations

TE: Transposable element
TRIM: Terminal inverted repeat in miniature
MITE: Inverted-repeat transposable element
AFLP: Amplified fragment length polymorphism
MSAP: Methylation-sensitive amplified polymorphism
TMD: Transposon methylation display
EST: Expressed sequence tag
NCBI: National center for biotechnology information
