# Supplementary material for: Structure and extent of DNA methylation-based epigenetic variation in wild emmer wheat (T. turgidum ssp. dicoccoides) populations
Source: BMC Plant Biol. 2015 Aug 14;15:200. doi: 10.1186/s12870-015-0544-z (PMC4536863; doi:10.1186/s12870-015-0544-z)
Supplement: Additional file 1: Table S1. — Ecogeographical data for five wild emmer wheat populations in Israel. [file 12870_2015_544_MOESM1_ESM.docx]

| **Collection site** | **Coordinates^1^** | | **Alt^2^** | **Soil type** | **Rn^3^** | **Temperature^4^** | | | |
| --- | --- | --- | --- | --- | --- | --- | --- | --- | --- |
|  | **Ln** | **Lat** |  |  |  | **Tm** | **Ta** | **Tj** | **Td** |
| Mt. Hermon | 35.76 | 33.29 | 1422 | Terra rossa | 1300 | 11 | 21 | 3 | 18 |
| Amiad | 35.53 | 32.92 | 240-350 | Terra rossa with limestone uneven micro-relief | 580 | 19 | 26 | 10 | 16 |
| Tabgha | 35.53 | 32.90 | 93 | Terra rossa | 436 | 24 | 32 | 15 | 17 |
| Jaba | 35.08 | 31.67 | 591 | Terra rossa | 500 | 17 | 25 | 9 | 15 |
| Mt. Aamasa | 35.11 | 31.35 | 814 | Soil pockets between large rocks micro-relief - brown lithosols and loessian arid brown soils | 271^5^ | 18^5^ | 25.3^5^ | 9.7^5^ | 16^5^ |

**Table S1**. Ecogeographical data for five wild emmer wheat populations in Israel (adapted from Nevo and Beiles 1989, Peleg *et al*. 2008 and Volis *et al*. 2015)

^1^Ln, longitude (decimal); Lat, latitude (decimal).

^2^Alt, altitude (m).

^3^Rn, annual rainfall (mm).

^4^Temperature (ºC): Tm, mean annual temperature; Ta, mean August temperature; Tj, mean January temperature; Td, seasonal temperature difference.

^5^ Data source: Israel Meteorological Service: <http://www.ims.gov.il/IMSEng/CLIMATE>.

**References:**

1. Nevo E, Beiles A: **Genetic diversity of wild emmer wheat in Israel and Turkey: structure, evolution and application in breeding.** *Theor Appl Genet* 1989, **77**:421-455.
2. [Peleg Z](http://www.ncbi.nlm.nih.gov/pubmed/?term=Peleg%20Z%5BAuthor%5D&cauthor=true&cauthor_uid=17908203), [Saranga Y](http://www.ncbi.nlm.nih.gov/pubmed/?term=Saranga%20Y%5BAuthor%5D&cauthor=true&cauthor_uid=17908203), [Krugman T](http://www.ncbi.nlm.nih.gov/pubmed/?term=Krugman%20T%5BAuthor%5D&cauthor=true&cauthor_uid=17908203), [Abbo S](http://www.ncbi.nlm.nih.gov/pubmed/?term=Abbo%20S%5BAuthor%5D&cauthor=true&cauthor_uid=17908203), [Nevo E](http://www.ncbi.nlm.nih.gov/pubmed/?term=Nevo%20E%5BAuthor%5D&cauthor=true&cauthor_uid=17908203), [Fahima T](http://www.ncbi.nlm.nih.gov/pubmed/?term=Fahima%20T%5BAuthor%5D&cauthor=true&cauthor_uid=17908203): **Allelic diversity associated with aridity gradient in wild emmer wheat populations.** *Plant Cell Environ* 2008, **31**(1):39-49.
3. Volis S, Ormanbekova D, Yermekbayev K, Song M, Shulgina I: **Multi-Approaches Analysis Reveals Local Adaptation in the Emmer Wheat (*Triticum dicoccoides*) at Macro- but not Micro-Geographical Scale.** *PLoS ONE* 2015, **10**(3): e0121153. doi: 10.1371/journal.pone.0121153
