# Supplementary material for: Structure and extent of DNA methylation-based epigenetic variation in wild emmer wheat (T. turgidum ssp. dicoccoides) populations
Source: BMC Plant Biol. 2015 Aug 14;15:200. doi: 10.1186/s12870-015-0544-z (PMC4536863; doi:10.1186/s12870-015-0544-z)
Supplement: Additional file 2: — Figure S1. Examples of MSAP banding patterns in wild emmer wheat accessions. (A) Radioactively-labeled MSAP patterns of three wild emmer wheat accessions. The DNA of each one of the accessions (samples) was cleaved either with the HpaII (H lane) or MspI (M lane) restriction enzyme. In each DNA sample, monomorphic bands between H and M lanes (black arrow) indicate that the CCGG site is unmethylated, while polymorphic bands (red arrow) indicate methylated sites. The methyl ation level for each accession is measured by dividing the total number of polymorphic sites (between H and M lanes) by the total number of sites. Note that monomorphic bands were scored only once. (B) Fluorescently-labeled MSAP patterns showing the H and M lanes of one of the wild emmer wheat accessions. The peak position (X axis) indicates the PCR product size of each band. The peak height (Y axis) indicates the band intensity, which has no merit in this qualitative analysis. The MSAP products were electrophoresed in a 3730xl DNA analyzer (Applied Biosystems) and analyzed using GeneMapper v4.0 (Applied Biosystems). All peak presence data were transferred to an excel file for further analysis. Figure S2. Average level of cytosine methylation in CCGG sites as assessed by MSAP in five wild emmer wheat populations (10 accessions in each population, indicated by diffirent colors). Standard errors are indicated. Figure S3. Non-metric Multi-Dimensional Scaling (MDS) anlaysis using the Jaccard similarity measure in Primer6 software for the MSAP analysis. MDS produces an ordination based on a distance or dissimilarity matrix where similar groups are clustered on a two dimensional plot. The index on the right top indicates the different groups (populations). The calculated p-values among the different groups are: 0.001 between Mt. Hermon and Amiad, 0.02 between Jaba and Amiad, 0.002 between Jaba and Mt. Hermon, 0.004 between Mt. Amasa and Amiad, 0.001 between Mt. Amasa and Mt. Hermon, 0.004 between Mt. Amas [file 12870_2015_544_MOESM2_ESM.docx]

**Supplemental Figures:**

**B.**

**A.**


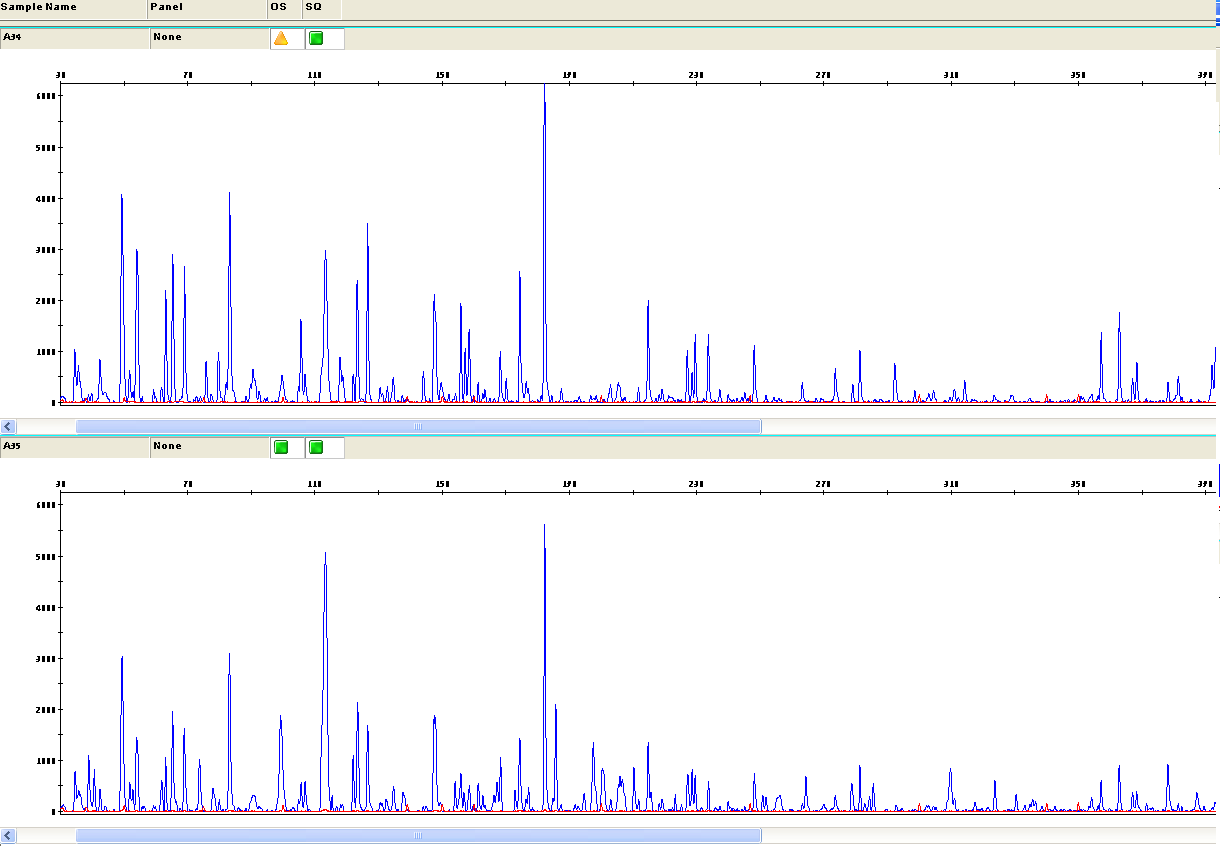


1 2 3

H M H M H M


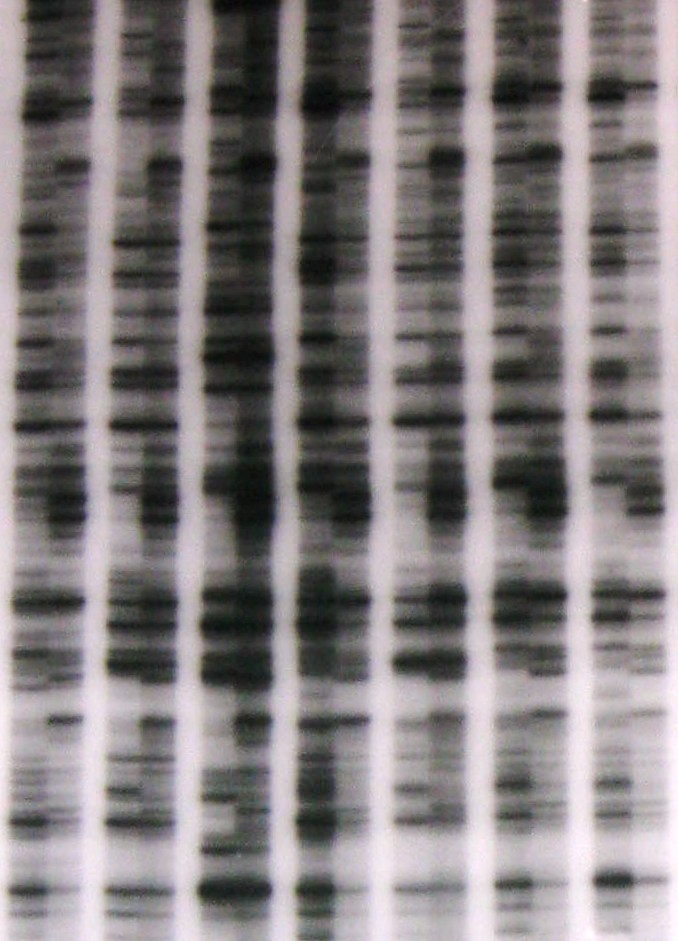


H lane

M lane

**Figure** **S1.** Examples of MSAP banding patterns in wild emmer wheat accessions. (**A**) Radioactively-labeled MSAP patterns of three wild emmer wheat accessions. The DNA of each one of the accessions (samples) was cleaved either with the *Hpa*II (H lane) or *Msp*I (M lane) restriction enzyme. In each DNA sample, monomorphic bands between H and M lanes (black arrow) indicate that the CCGG site is unmethylated, while polymorphic bands (red arrow) indicate methylated sites. The methylation level for each accession is measured by dividing the total number of polymorphic sites (between H and M lanes) by the total number of sites. Note that monomorphic bands were scored only once. (**B**) Fluorescently-labeled MSAP patterns showing the H and M lanes of one of the wild emmer wheat accessions. The peak position (X axis) indicates the PCR product size of each band. The peak height (Y axis) indicates the band intensity, which has no merit in this qualitative analysis. The MSAP products were electrophoresed in a 3730xl DNA analyzer (Applied Biosystems) and analyzed using GeneMapper v4.0 (Applied Biosystems). All peak presence data were transferred to an excel file for further analysis.

**Figure S2.** Average level of cytosine methylation in CCGG sites as assessed by MSAP in five wild emmer wheat populations (10 accessions in each population, indicated by diffirent colors). Standard errors are indicated.


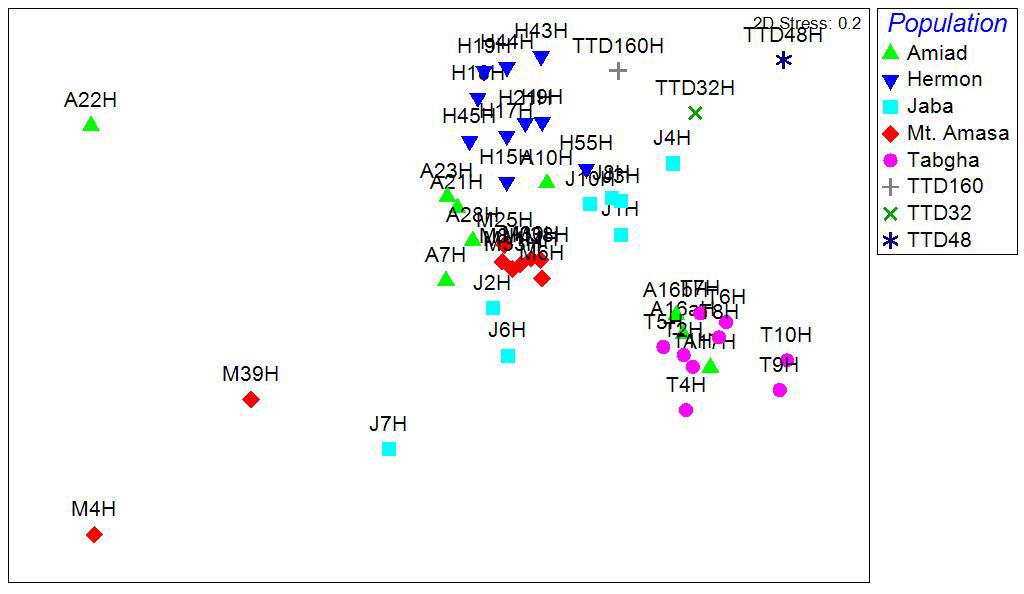
**Figure S3.** Non-metric Multi-Dimensional Scaling (MDS) anlaysis using the Jaccard similarity measure in Primer6 software for the MSAP analysis. MDS produces an ordination based on a distance or dissimilarity matrix where similar groups are clustered on a two dimensional plot. The index on the right top indicates the different groups (populations). The calculated *p*-values among the different groups are: 0.001 between Mt. Hermon and Amiad, 0.02 between Jaba and Amiad, 0.002 between Jaba and Mt. Hermon, 0.004 between Mt. Amasa and Amiad, 0.001 between Mt. Amasa and Mt. Hermon, 0.004 between Mt. Amasa and Jaba, 0.004 between Tabgha and Amiad, 0.001 between Tabgha and Mt. Hermon, 0.004 between Tabgha and Jaba, and 0.004 between Tabgha and Mt. Amasa.

**Figure S4.** Average level of cytosine methylation in CCGG sites flanking *Veju* retrotransposon as assessed by TMD in five wild emmer wheat populations (10 accessions in each population, indicated by diffirent colors). Standard errors are indicated.


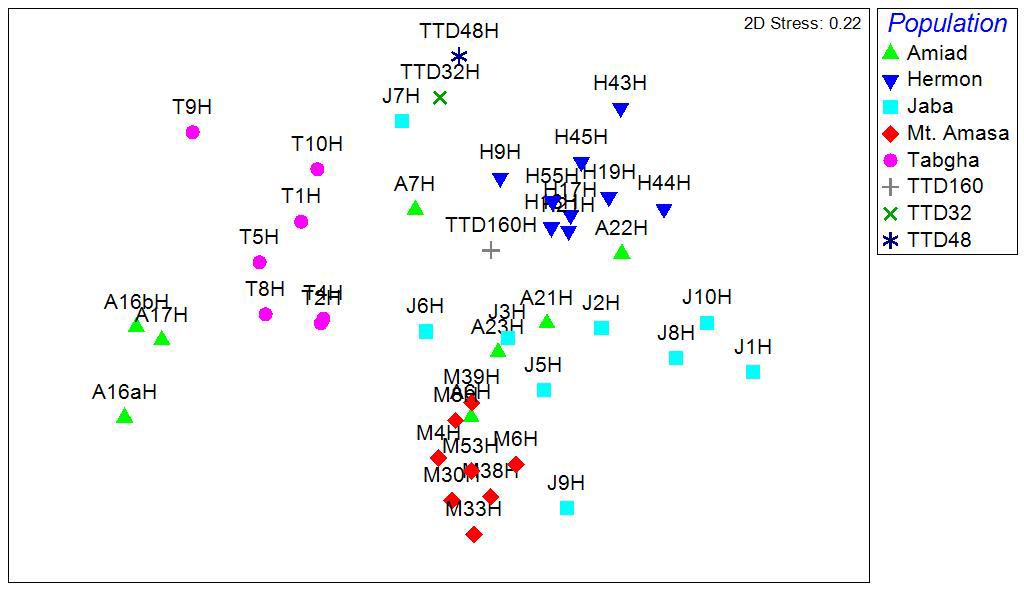


**Figure S5.** Non-metric Multi-Dimensional Scaling (MDS) anlaysis using the Jaccard similarity measure in Primer6 software for TMD analysis of *Veju* retrotransposon. The index on the right top indicates the different groups (populations). The calculated *p*-values among the different groups are: 0.004 between Mt. Hermon and Amiad, 0.1 between Jaba and Amiad, 0.004 between Jaba and Mt. Hermon, 0.03 between Mt. Amasa and Amiad, 0.004 between Mt. Amasa and Mt. Hermon, 0.008 between Mt. Amasa and Jaba, 0.2 between Tabgha and Amiad, 0.009 between Tabgha and Mt. Hermon, 0.009 between Tabgha and Jaba, and 0.02 between Tabgha and Mt. Amasa.

**Figure S6.** Average level of cytosine methylation in CCGG sites flanking *Thalos* DNA-transposon as assessed by TMD in five wild emmer wheat populations (10 accessions in each population, indicated by diffirent colors). Standard errors are indicated.


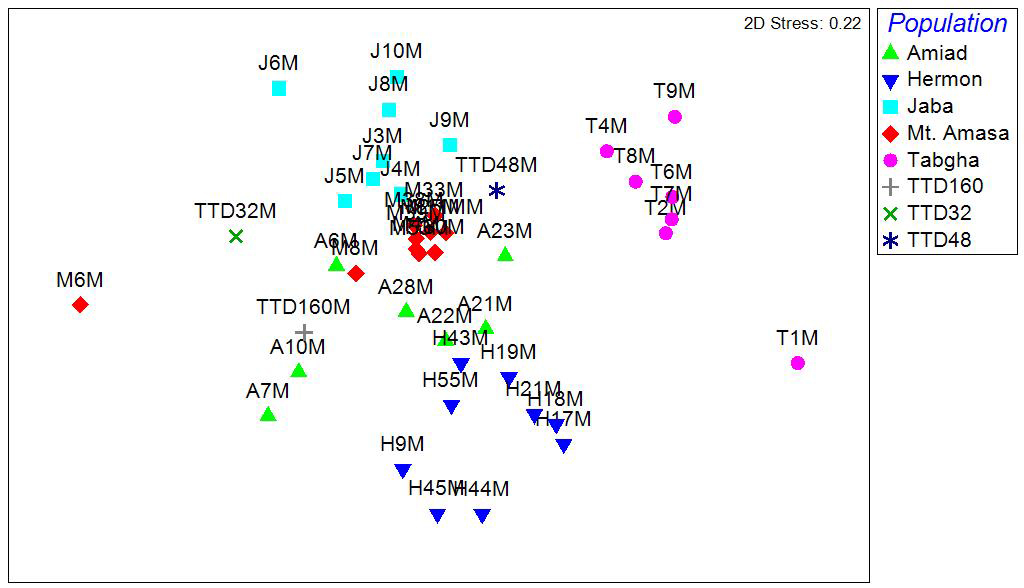


**Figure S7.** Non-metric Multi-Dimensional Scaling (MDS) anlaysis using the Jaccard similarity measure in Primer6 software for TMD analysis of *Thalos* DNA transposon. The index on the right top indicates the different groups (populations). The calculated *p*-values among the different groups are: 0.009 between Mt. Hermon and Amiad, 0.02 between Jaba and Amiad, 0.004 between Jaba and Mt. Hermon, 0.1 between Mt. Amasa and Amiad, 0.001 between Mt. Amasa and Mt. Hermon, 0.02 between Mt. Amasa and Jaba, 0.06 between Tabgha and Amiad, 0.009 between Tabgha and Mt. Hermon, 0.02 between Tabgha and Jaba, and 0.005 between Tabgha and Mt. Amasa.
